# Severe summer heatwave and drought strongly reduced carbon uptake in Southern China

**DOI:** 10.1038/srep18813

**Published:** 2016-01-07

**Authors:** Wenping Yuan, Wenwen Cai, Yang Chen, Shuguang Liu, Wenjie Dong, Haicheng Zhang, Guirui Yu, Zhuoqi Chen, Honglin He, Weidong Guo, Dan Liu, Shaoming Liu, Wenhua Xiang, Zhenghui Xie, Zhonghui Zhao, Guomo Zhou

**Affiliations:** 1 State Key Laboratory of Earth Surface Processes and Resource Ecology, Beijing Normal University, Beijing 100875, China; 2 State Key Laboratory of Cryospheric Sciences, Cold and Arid Regions Environmental and Engineering Research Institute, Chinese Academy of Sciences, Lanzhou 730000, Gansu, China; 3 National Engineering Laboratory for Applied Technology of Forestry & Ecology in South China, Central South University of Forestry and Technology, Changsha 410004, Hunan, China; 4 Key Laboratory of Ecosystem Network Observation and Modeling, Synthesis Research Center of Chinese Ecosystem Research Network, Institute of Geographic Sciences and Natural Resources Research, Chinese Academy of Sciences, Beijing 100101, China; 5 College of Global Change and Earth System Science, Beijing Normal University, Beijing 100875, China; 6 Institute for Climate and Global Change Research & School of Atmospheric Sciences, Nanjing University, China; 7 State Key Laboratory of Remote Sensing Science, School of Geography, Beijing Normal University, Beijing 100875, China; 8 State Key Laboratory of Numerical Modeling for Atmospheric Sciences and Geophysical Fluid Dynamics, Institute of Atmospheric Physics, Chinese Academy of Sciences, Beijing, China; 9 Zhejiang Agriculture and Forestry University, Lin’an 311300, China

## Abstract

Increasing heatwave and drought events can potentially alter the carbon cycle. Few studies have investigated the impacts of hundred-year return heatwaves and droughts, as those events are rare. In the summer of 2013, southern China experienced its strongest drought and heatwave on record for the past 113 years. We show that the record-breaking heatwave and drought lasted two months (from July to August), significantly reduced the satellite-based vegetation index and gross primary production, substantially altered the regional carbon cycle, and produced the largest negative crop yield anomaly since 1960. The event resulted in a net reduction of 101.54 Tg C in carbon sequestration in the region during these two months, which was 39–53% of the annual net carbon sink of China’s terrestrial ecosystems (190–260 Tg C yr^−1^). Moreover, model experiments showed that heatwaves and droughts consistently decreased ecosystem vegetation primary production but had opposite impacts on ecosystem respiration (TER), with increased TER by 6.78 ± 2.15% and decreased TER by 15.34 ± 3.57% assuming only changed temperature and precipitation, respectively. In light of increasing frequency and severity of future heatwaves and droughts, our study highlights the importance of accounting for the impacts of heatwaves and droughts in assessing the carbon sequestration in terrestrial ecosystems.

As a consequence of climate change, the incidence and severity of heatwaves and droughts have substantially increased since the middle of the 20th century^1^. The frequency of extreme heatwave events increased significantly for over 73% of the global land area^2^,^3^, and the number of occurrences has close to doubled in Europe, Australia and much of Asia^4^,^5^. Extensive severe drought episodes are frequently reported across the globe^6^. Recent large-scale and severe droughts have occurred in Europe in 2003^7^, western North America from 1999 to 2004^8^, and Northeast China from the 1999 to 2011^9^.

Carbon and hydrological cycles strongly couple in terrestrial ecosystems; therefore, such extreme climate events substantially impact terrestrial carbon cycle dynamics as well as atmospheric carbon dioxide concentrations. A recent study quantified the spatiotemporal contiguous extreme anomalies in four global datasets of gross primary production (GPP) during 1982–2011 and found that the largest thousand negative GPP extremes account for a decrease in photosynthetic carbon uptake of approximately 3.5 Pg C yr^−1^, with most events being attributable to water scarcity^10^. Numerous studies evaluated the impacts of drought on ecosystem carbon cycles globally^11^,^12^. For example, in Northern China, the multiyear precipitation reduction changed the regional carbon uptake of 0.011 Pg C yr^−1^ from 1982 to 1998 to a net source of 0.018 Pg C yr^−1^ from 1999 to 2011, and the average maize yield from 1999 to 2011 was reduced by 440 kg ha^−1^ yr^−1^ compared with linear trend yields^13^.

Droughts often occur accompanied by severe heatwaves, which together generate combined effects on carbon cycles. When combined with heat, drought tends to exacerbate heat responses by reducing transpirational cooling and increasing leaf temperature^14^,^15^. Some studies suggested that heatwaves might only stimulate plant growth when soil moisture is plentiful and temperatures are suboptimal (outside of summer)^16^. In contrast, several studies also highlighted that heat enhances drought because of faster soil drying^17^. Meanwhile, heatwaves can induce high vapor pressure deficits, which inhibit stomatal opening. This indirect effect of heat may have a larger impact on plants than warming itself^18^.

Ecosystem carbon models have often been used as a tool to investigate the effects of climate extremes on ecosystem carbon cycling 19. Ecosystem models can quantify the magnitude of impacts on ecosystems carbon cycles resulting from extreme events from several limited sites to other ecosystems, wider geographic areas and into the future. Therefore, in the past few decades, many ecosystem models have been used to estimate the intensity and extensity of climate extremes over the regional and global scales^7^,^8^,^12^,^13^. For example^7^,) used a process-based ecosystem model (ORCHIDEE biosphere model) to estimate the Europe-wide impacts of the anomalous 2003 climate, and the model was first verified at eddy-covariance sites and then simulated the Europe-wide changes in carbon fluxes using reconstructed climate and weather analyses. Moreover, ecosystem models are designed to reflect the general understanding of ecosystem processes according to scientific communities and can provide insights into mechanisms of impacts on ecosystem processes.

Few studies, however, have investigated the impacts of hundred-year return heatwaves and droughts, as those events are rare. During the summer of 2013, southern China, including 9 provinces and 2 provincial municipalities, experienced the worst drought and heatwave during the past 113 years, with the highest area-averaged air temperature and lowest precipitation and relative humidity according to historical records. In this study, the overarching goal is to assess the impacts of this hundred-year return heatwave and drought on the terrestrial carbon balance. The specific objectives are to (1) evaluate the severity of the heatwave and drought in the summer of 2013 across southern China, and (2) investigate the impacts of the heatwave and drought on the carbon budget.

## Data and Methods

### Study area and data

**Figure 1:**
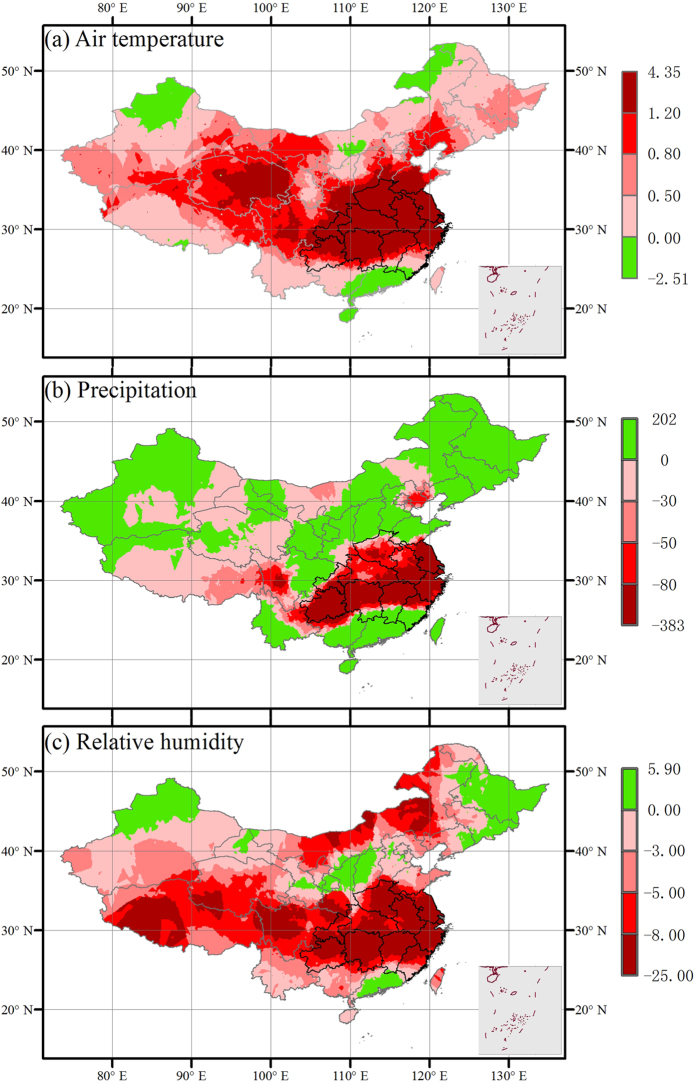
Regional anomalies of air temperature (^o^C) (**a**), precipitation (mm) (**b**) and relative humidity (%) (**c**) during July-August 2013. All data compare 2013 and the average of 1960–2012. The provinces with bold black boundary lines are the study area in this study. The right-bottom figures show the boundary of South China Sea. The maps were created by the ArcMap 9.3.

The study area includes 9 provinces and 2 provincial municipalities in southern China (Fig. 1, S1), covering 146.68 million km^2^ and accounting for 15.28% of the territory of China. Forests, croplands and grasslands are the dominant vegetation types in the study area, including 26.09% forests, 27.30% croplands, and 8.87% grasslands in China. This area plays an important role in crop production and carbon storage in China.

To investigate whether anomalies in the study area, we collected daily meteorological data from 1960 to 2013 from 756 stations from the National Climate Center of the Chinese Meteorological Administration. A total of 191 stations were located within the study area (Fig. S1). To identify the magnitude of the heatwave and drought in the summer of 2013, especially from the hundred-year perspective, we combined observations of air temperature, precipitation and relative humidity at all meteorological sites with the CRU (Climatic Research Unit) dataset covering from 1900 to 2012. The CRU TS 3.0 climate dataset was obtained from the Climatic Research Unit at the University of East Anglia (http://www.cru.uea.ac.uk/data). This gridded dataset, with a spatial resolution of 0.5° × 0.5°, was based on climate observations from more than 4,000 meteorological stations.

Thin plate smoothing splines were used to produce the daily mean temperature, maximum and minimum air temperature, precipitation, relative humidity, sun shine duration, wind speed and atmospheric pressure for the entire China territory, with a spatial resolution of 25 × 25 km^20^; the data were used to drive the ecosystem models for examining the impacts of weather anomalies on the regional carbon cycle.

The Normalized Difference Vegetation Index (NDVI) from the Terra satellite’s Moderate Resolution Imaging Spectroradiometer (MODIS) is a composite of leaf area and chlorophyll content. MODIS NDVI products (MOD13) during 2000–2013 were used to investigate the responses of vegetation to drought and heatwave conditions. A method based on Savitzky–Golay filter^21^ was used to smooth out noise in the NDVI time-series, primarily cloud contamination and atmospheric variability.

The presence of a network of instrumentation using eddy covariance towers for the monitoring of ecosystem fluxes at this time, with continuous records of CO_2_, helped us to assess the impact of such an extreme heatwave and drought event on the carbon balance. This study includes six eddy covariance (EC) sites in the study area (Table 1), covering grasslands and forests, to investigate the impacts of droughts and heatwaves and examine the performance of the EC-LUE (Eddy Covariance – Light Use Efficiency) and IBIS (Integrated BIosphere Simulator) models (see below) in reproducing the gross primary production (GPP), ecosystem respiration (TER), and net ecosystem production (NEP). The eddy covariance flux measurements were collected using a Gill Sonic anemometer (Model R3; Gill Instruments Ltd, Lymington, UK), a closed-path system (LI-6262), and the LI-7500 open-path CO_2_/H_2_O water vapor sensor (LI-Cor, Lincoln, NE). The key supporting meteorological variables that were measured included air temperature, humidity, photosynthetically active radiation, precipitation, soil heat flux, solar radiation, net radiation, and windspeed. Due to limited data sharing policy, we only used the EC measurements before August of 2013.

The EC data analysis procedures followed those of Reichstein *et al.*^22^ and Yuan *et al.*^23^. The partitioning between GPP and terrestrial ecosystem respiration was completed according to the method proposed in Reichstein *et al.*^22^. Eddy covariance systems directly measure net ecosystem exchange (NEE) rather than GPP. To estimate GPP, it is necessary to estimate daytime respiration (R_d_):


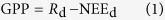


where NEE_d_ is daytime NEE. Daytime ecosystem respiration R_d_ is usually estimated by using daytime temperature and an equation describing the temperature dependence of respiration, which is subsequently developed from nighttime NEE measurements. Nighttime NEE represents nighttime respiration because plants do not photosynthesize at night. The following model^24^ was used to describe the effects of temperature on night-time NEE:


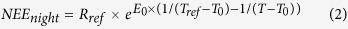


where NEE_night_ is the night-time ecosystem respiration, and T is the night-time air temperature. The regression parameter T_0_ was kept constant at −46.02 °C, as in Lloyd and Taylor^24^, and the reference temperature (T_ref_) was set to 10 °C, as in the original model. The parameters E_0_ (activation energy) and R_ref_ (reference ecosystem respiration) were determined using nonlinear optimization. Eq. (2) and daytime temperature were subsequently used to estimate daytime respiration (R_d_). Meanwhile, Eq. (2) was applied to fill the missing nighttime fluxes.

A Michaelis–Menten type light response function was used to fill the missing daytime fluxes (NEE_day_)^25^


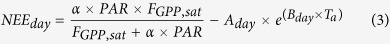


where F_GPP,sat_ (gross primary productivity at saturating light), α (initial slope of the light response function), T_a_ is the air temperature of daytime, PAR is photosynthetically active radiation, A_day_ and B_day_ are fitted parameters. The nonlinear regression procedure (PROC NLIN) in the Statistical Analysis System (SAS Institute Inc., Cary, NC, USA) was used to fit the relationships between measured fluxes and environmental factors. Regression relationships between measured fluxes and meteorological conditions were fitted using a 7-day moving window. In this study, no attempt was made to fill the data gaps of environmental variables (i.e. air temperature and PAR), and the data gaps of carbon fluxes cannot be filled when environmental variables were missing. Daily GPP, TER and NEP were synthesized based on half-hourly values and the daily values were indicated as missing when missing data was 20% of entire data at a given day, otherwise daily values were calculated by multiplying averaged half-hourly rate by 24 (hours). If missing daily data were > 20% of the entire year, then the value of this year was not calculated.

Mean harvest-yield of several crops (i.e. wheat, maize and rice) data from province-level statistics from 1960–2013 were used to analyze drought impacts (http://data.stats.gov.cn, National Bureau of Statistics of China). Crop yield is influenced by various biotic, abiotic and anthropogenic factors (e.g., environment and management) and shows trends due to improvements in genetics, and fertilizer application policies. To remove the impacts of improved agriculture and reveal the influence of climate, the crop yield time series were de-trended using the best fit least squares regression method as recommended by Goldblum^26^. The linear regression was used in this study to calculate the expected yield, and residual values were calculated as deviations from the expected yield and observed yield value. The residual values indicate the impacts of climate change.

### Model experiments

In this study, a satellite-based light use efficiency (LUE) model (EC-LUE, Eddy Covariance Light Use Efficiency^27^,^28^,^29^) and an ecosystem physiological model (IBIS, Integrated Biosphere Simulator; 30), which have been widely validated and applied at global scales, were used to examine the changes in vegetation gross primary productivity (GPP), and the latter also simulated ecosystem respiration (TER) and net ecosystem production (NEP) to investigate the impacts of the anomalous 2013 climate in southern China. First, we examined the model ability to reproduce the observed GPP, TER and NEP anomalies at multiple eddy-covariance sites within the study area and other regions with similar climate conditions globally (Supplementary Table S1). Second, we simulated the regional changes in carbon fluxes from 1960 to 2013. Detailed modeling methods are provided in the supplementary material.

To differentiate the individual and confounding effects of heatwaves and droughts, or more specifically the three factors (i.e., high temperature, low precipitation and low relative humidity), on carbon uptake, we conducted modeling experiments using IBIS and various combinations of driving datasets according to a factorial design. We used the long-term (1960–2012) averaged monthly temperature, precipitation and relative humidity to replace the driving data during July and August of 2013 to represent the normal conditions (i.e., the control model experiment). The individual impacts of high temperature (HT), low precipitation (LP), and relative humidity (LR) were investigated by replacing the other two variables using long-term monthly averages. Similarly, three two-factor modeling experiments were conducted on top of the normal conditions: (1) increased temperature and decreased precipitation (HT + LP), (2) increased temperature and decreased relative humidity (HT + LR), and (3) decreased precipitation and decreased relative humidity (LP + LR). For example, in the HT + LP experiment, the actual temperature and precipitation data of July and August of 2013 were used to drive the model, with the long-term mean relative humidity. The overall impacts of heatwaves and droughts were the combined impacts of three factors: increased temperature, decreased precipitation and decreased relative humidity (HT + LP + LR). The percentage changes in GPP, TER, NEP and soil water content (Ws) relative to the control experiment were calculated and evaluated.

## Results

### Heatwaves and droughts in southern China

**Figure 2:**
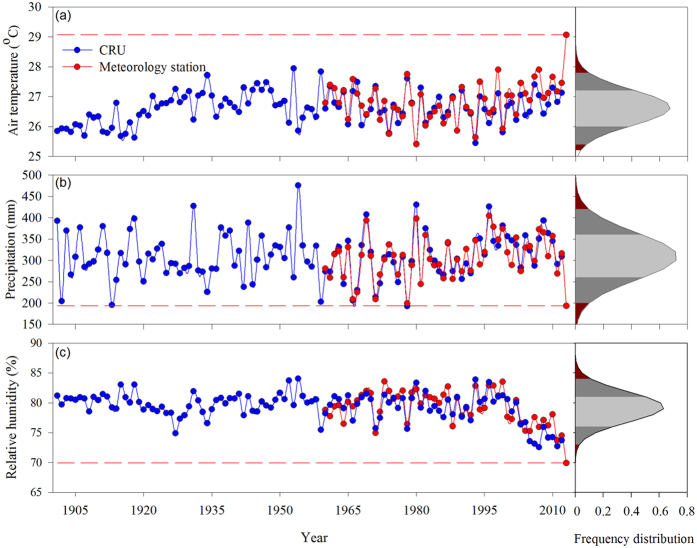
Observed air temperature, precipitation and relative humidity of July and August during the past 113 years. (**a**) area-averaged air temperature based on CRU dataset (blue lines) and 191 meteorology sites dataset (red lines). (**b,c**) same for precipitation and relative humidity. Dashed red horizontal lines indicate the values of 2013. The right panes showed the frequency distribution of three variables. The dark red indicate the area out of the 95^th^ percentile range (2 standard deviation), and the dark gray indicate the 68^th^ percentile range (1 standard deviation).

Mean air temperature from all standard meteorological stations in the region reported new historical highs, and the regional temperature in July and August was 4.35 °C higher than the long-term mean from 1960 to 2012. Based on the probability distribution function (Fig. 2a), the heatwave of July and August in 2013 only has 0.0003% chance of occurring in any one year. Total precipitation of these two months decreased 383 mm or 78% of the average amount (Figs 1b and 2b), breaking the historical record (Fig. 2b). The heatwave and drought together led to substantial decreases in relative humidity, with average relative humidity declining 25% and reaching a record low (Figs 1c and 2c).

### Impacts of heatwaves and droughts on carbon uptake

**Figure 3:**
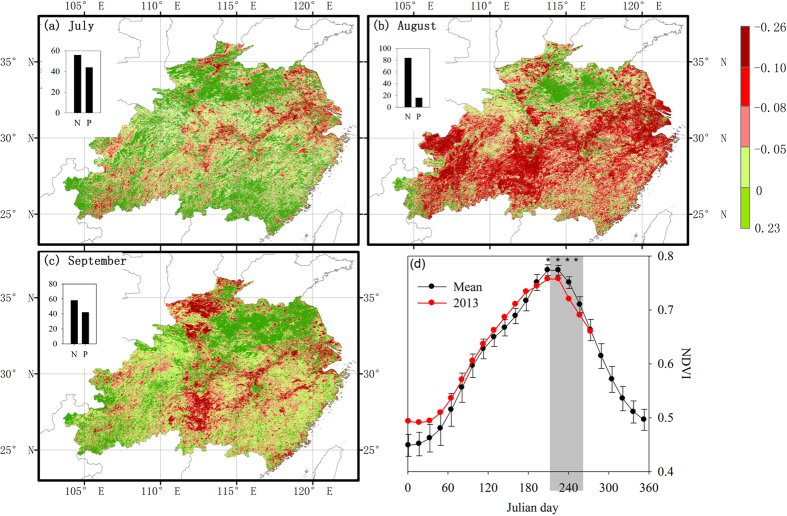
Anomalies of NDVI during July (**a**), August (**b**), September (**c**) of 2013, and area-average NDVI (**d**). All data compare 2013 and the mean values of 2000–2012. The panels in (**a**–**c**) show the percentage (%) of the pixels with positive anomalies (P, green bars) and negative anomalies (N, red bars). The grey area indicates the period of July and August, and *show the significant differences of NDVI between 2013 and mean values of 2000–2012. The maps were created by the ArcMap 9.3.

Before the start of the drought (i.e., June 2013), only 18% of the vegetated area in the region exhibited negative NDVI anomalies and regional average NDVI was higher than the mean from 2000 to 2012 (Fig. 3d). During the drought, however, the areas of negative NDVI anomalies expanded significantly to 56% and 84% of the total vegetated area (Fig. 3a,b,d) in July and August, respectively. The regional NDVI of August 2013 reached the lowest record for that month since the start of the MODIS observations (i.e., 2000) (Fig. S2). Moreover, vegetation did not entirely recover from the heat-drought stress during the subsequent September, as negative NDVI anomalies were still found in 58% of the area (Fig. 3c,d).

**Figure 4:**
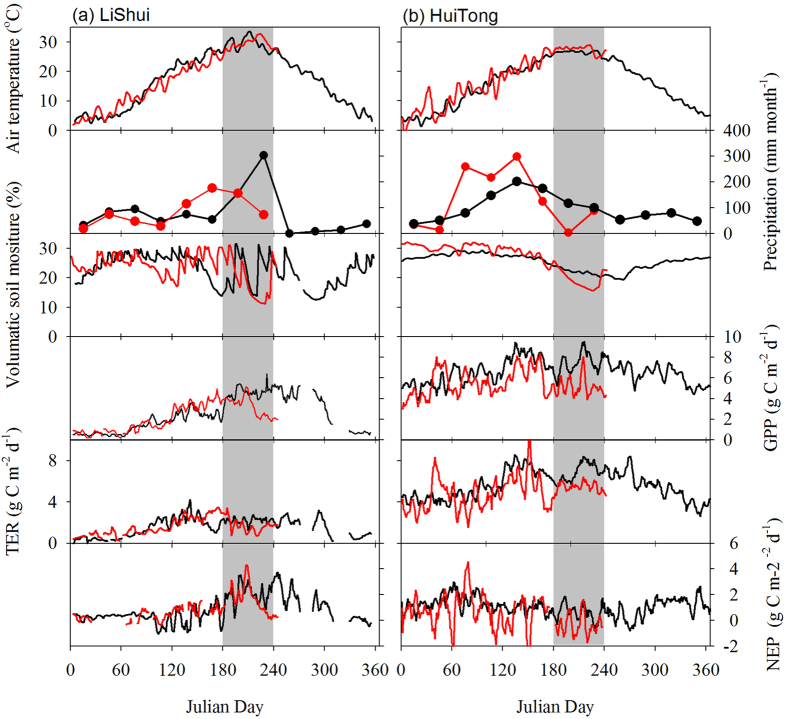
Observed climate and ecosystem CO_2_ fluxes at two eddy covariance sites, LiShui (**a**) and HuiTong (**b**). A five day running average was applied to the original half-hourly flux. Precipitation values are monthly averages. Red lines indicate the data of 2013, and black lines indicate 2012 at LiShui and mean values from 2009–2012 at HuiTong. The July to August period is shaded in grey.

All six sites (i.e., 5 forests and 1 grassland) monitored with eddy covariance instrumentation experienced a substantial GPP reduction in July and August of 2013 (Table 2). On average, the GPP at all 6 sites decreased by 47.74 g C m^−2^ month^−1^, ranging from 42.47 to 55.49 g C m^−2^ month^−1^, compared with those in July and August of the baseline period (see Table 2), which was quite similar to the simulated decrease in magnitude by EC-LUE (52.08 g C m^−2^ month^−1^) and IBIS (53.01 g C m^−2^ month^−1^). The GPP drop coincided with reduced soil moisture due to the rainfall deficit (Table 2; Fig. 4). Total ecosystem respiration (TER) decreased at all flux tower sites during the heat-drought event (Table 2). GPP was more sensitive to heat-drought than TER, and a larger reduction of GPP than TER led to anomalous carbon sources at all flux tower sites (Table 2). The drought and heat wave during July and August substantially impacted carbon uptake at nearly all sites in 2013. On average, NEP decreased by −34.41 g C m^−2^ month^−1^ over all study sites during July and August of 2013 compared to the baseline period (Table 2).

**Figure 5:**
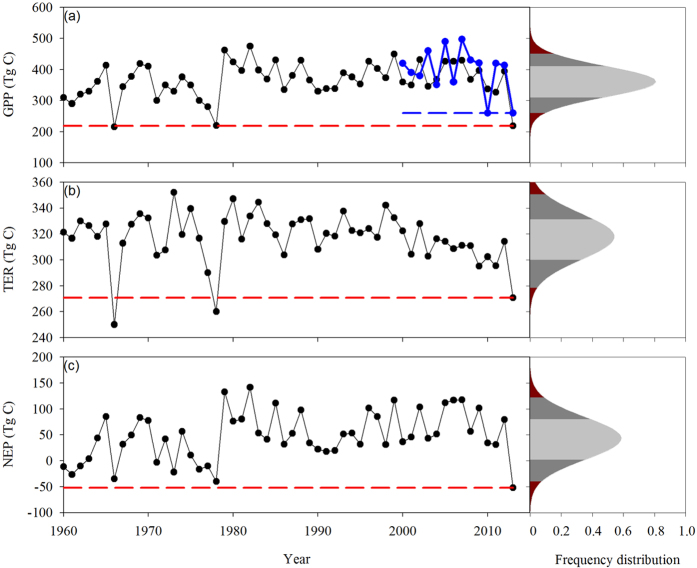
Interannual variability of simulated area-averaged vegetation gross primary production (GPP) from IBIS and EC-LUE (**a**), ecosystem respiration (TER) (**b**) and net ecosystem production (NEP) (**c**) during July and August. (**a**) Black line indicates the IBIS simulations from 1960 to 2013 and blue line indicates EC-LUE simulations from 2000 to 2013. The dashed lines indicate the value of 2013. The right panes showed the frequency distribution of three variables. The dark red indicate the area out of the 95^th^ percentile range (2 standard deviation), and the dark gray indicate the 68^th^ percentile range (1 standard deviation).

Regional GPP was reduced 146.96 Tg C and 149.59 Tg C according to EC-LUE and IBIS simulations, respectively, during July and August of 2013 over the study area (146.68 million km^2^), representing 36.12% and 40.52% reductions, respectively, compared to the long-term averaged GPP values from 2000 to 2012 for EC-LUE and from 1960 to 2012 for IBIS (Fig. 5a). These simulations were consistent with the estimated GPP reduction from eddy-covariance observations (Table 2). Simulated TER by IBIS fell in July and August of 2013 by 47.45 Tg C compared with the long-term average from 1960 to 2012. Apparently, the TER reduction was smaller than the GPP reduction, again consistent with observations at the eddy-covariance sites (Table 2). We found that July and August of 2013 had the lowest net ecosystem production (NEP) during the entire IBIS simulation period, and the drought and heatwave changed the regional carbon budget from carbon uptake of 49.44 Tg C, averaged from 1960 to 2012, to a net source of 52.10 Tg C during these two months. This finding suggests that the two-month-long drought decreased carbon sequestration of 101.54 Tg C, which was 39–53% of the annual net carbon sink of China’s terrestrial ecosystems (190–260 Tg C yr^−1^)^31^.

**Figure 6:**
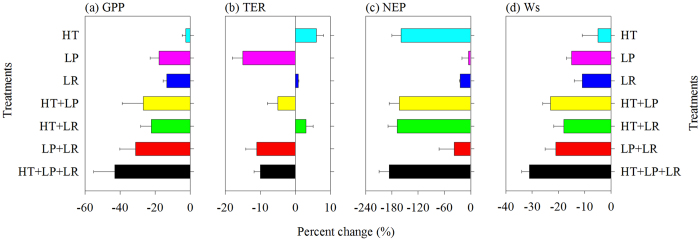
Percent changes relative to control in modeled gross primary production (GPP) (**a**), ecosystem respiration (TER) (**b**), net ecosystem production (NEP) (**c**) and soil water content (Ws) (**d**) in response to treatments of high temperature only (HT), decreased precipitation only (LP), decreased relative humidity only (LR) and joint impacts of two and three variables. Data indicate the mean ± SE calculated from simulated values over the entire study areas.

Moreover, we conducted model experiments to differentiate the individual and confounding effects of heatwave and drought conditions on carbon uptake (see Method). IBIS simulations showed that GPP was consistently reduced under increased temperature (HT), decreased precipitation (LP), and decreased relative humidity (LR), and the decreased magnitude under LP (17.79 ± 5.21%) and LR (13.34 ± 2.13%) was comparable (Fig. 6a). Together, they resulted in a severe decrease in GPP (43.27 ± 12.08%, HT + LP + LR). The overall response pattern of the modeled soil water content (Ws) to the changes in these three driving forces was similar to that of GPP (Fig. 6d), suggesting that changes in Ws might be the major direct cause for the suppressed GPP. Separately, heatwave (HT) and drought (LP) had opposite impacts on TER, increasing TER by 6.78 ± 2.15% and decreasing TER by 15.34 ± 3.57%, respectively (Fig. 6b). Heatwave and drought conditions together decreased TER by 10.35 ± 1.98% due to the larger impact of drought than that of heatwave conditions. In comparison with the normal conditions, heatwave conditions alone or together with drought can lead to a 159–186% reduction in modeled NEP (Fig. 6c). Apart from the heatwave effects, drought (low precipitation and relative humidity) concurrently depressed the GPP and TER, which offset its impacts on NEP. Our simulations implied the important role of high temperature accompanied by drought on terrestrial ecosystem carbon uptake.

**Figure 7:**
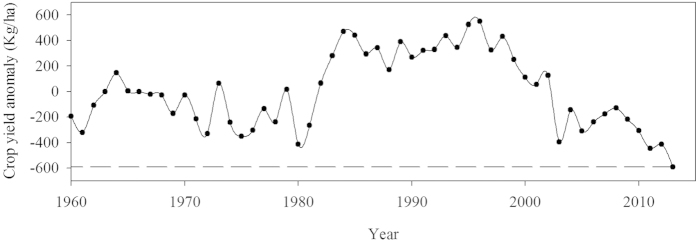
Observed changes of crop yield anomaly from 1960 to 2013. A linear trend has been removed from the data to subtract the effects of improved agriculture and let appear the climate-induced variability.

The change in crop yield in 2013 can be another indicator to measure the impacts of drought conditions on ecosystem productivity and carbon cycle dynamics. Decreases in summer crop yield were reported across most of the provinces and municipalities in the region, with the averaged crop yield decreasing by 90.91 kg ha^−1^ (data not shown). The harvest data, after de-trending, indicated that 2013 was the year with the largest negative crop yield anomaly (−591 kg ha^−1^) during the past 53 recorded years (Fig. 7). The chance of observing such a reduction in crop yield was 1.22% (an 83-year return event).

## Discussion

### Droughts and heatwaves in southern China

Abundant precipitation and warm temperature in southern China support extensive forests and croplands, which play important roles in carbon uptake and the food supply. Ecosystems and their services in the region are increasingly affected by frequent heatwaves and droughts^32^,^33^. However, none of the heatwaves and droughts of the past recorded 113 years has surpassed the one observed in 2013 in severity and extent. Covering at least 9 provinces and 2 provincial municipalities in the region, the protracted 2013 heatwave and drought lasted two months, from July to August, and is a hundred-year return event.

This study, using multiple lines of evidence, has demonstrated that a hundred-year return heat and drought event in the summer of 2013 significantly altered the regional carbon cycle, and its impacts at the continental level and in the long run have not been evaluated. The importance of droughts and heatwaves will likely increase in the region and across the continent in light of the projected increasing magnitude and frequency of droughts in Southern China within the next several decades^34^,^35^. It has been suggested that El Niño-like warming in the tropical Pacific could lead to weakened summer monsoons and thus severe drought in East China^36^. According to the multimodel predictions of CMIP5 models, southern China is one of the major areas with substantial decreases in soil-moisture content in the top-10-cm layer within the next several decades^34^. Similar changes are also seen in the predictions of the CMIP3 model and the Palmer Drought Severity Index (PDSI), and the PDSI suggests stronger decreases in soil moisture^34^. More frequent extreme drought events may counteract the effects of the anticipated mean warming and lengthening of the growing season^37^ and erode the health and productivity of ecosystems, reversing sinks to sources and contributing to positive carbon-climate feedbacks. For example, a previous study showed that drought-induced reduction in summer photosynthesis cancels out the CO_2_ uptake enhancement induced by warmer springs in the northern hemisphere^17^.

### Differential impacts of droughts and heatwaves on the carbon cycle

Numerous studies have revealed the impacts of drought on vegetation production. Drought is known to inhibit cell expansion and reduce stomatal opening and carbohydrate supply, thus impeding growth and productivity^38^. Widespread droughts can induce large-scale tree decline episodes in terrestrial ecosystems^39^ and turn ecosystems into carbon sources, contributing to positive carbon-climate feedback^8^. For example, in Amazonian rainforests, the 2005 drought decreased plant production in association with increased mortality and reduced growth^40^.

High temperature can impact vegetation production by increasing the vapor pressure deficit (VPD) and decreasing soil moisture. Previous studies found that strongly positive correlations dominate the relationship between air temperature and VPD and suggest that summer heatwaves can induce high VPD^16^. Field control experiments also showed that mean daytime VPD immediately doubled when temperatures increased 4 °C^41^. High VPD can substantially decrease leaf stomatal conductance and constrain canopy-level CO_2_ uptake^42^,^43^,^44^. Lower CO_2_ uptake by the canopy may have led to decreased canopy development and duration and thus to lower NPP. This VPD response may have been exacerbated by drier surface soils in warmed ecosystems, potentially lowering the apparent stomatal conductance.

Warmer temperatures are expected to increase both microbial and plant respiration. In this study, model experiments suggested that high temperature stimulated ecosystem respiration, and other studies support our conclusion. For example, a meta-analysis showed that soil respiration was stimulated by 9.0% under manipulative warming, with a 9.4% increase in autotrophic respiration and a 7.5% increase in heterotrophic respiration. In addition, ecosystem respiration significantly increased by 6.0%^45^.

However, under the combined effects of high temperature and low precipitation, total ecosystem respiration (TER) decreased at all flux tower sites during the heat-drought event (Table 2). The exact mechanisms behind the decline of TER during the heat-drought event are not clear but might be multifaceted. First, plant autotrophic respiration (Ra) is probably reduced, as it is proportionally related to the suppressed GPP^46^. Second, numerous studies showed that high temperature and drought will decrease microbial biomass and enzyme activities, which will suppress the soil respiration. For example, an incubation experiment showed that soil respiration and enzyme activities increased upon temperature increases up to 30–55 °C and then decreased in all soils after exposure at higher temperatures^47^. Soil moisture plays an important role in regulating soil microbial biomass, respiration and enzyme activity^48^, and stronger soil respiration and enzyme activity have been reported in wet soil than dry soil in both laboratory and field studies^49^. Third, although increased temperature can enhance heterotrophic respiration (Rh), this impact can be reduced or overpowered by the simultaneous impact of water limitation according to the Libig law (i.e., the rate of a biological process is controlled by the most limiting factor), resulting in an overall reduction in Rh. Other lines of evidence support our result that ecosystem respiration decreases with soil moisture stress;^8^,^13^,^50^. Drought decreased plant photosynthesis and maintenance respiration because of depressed plant physiological processes^46^. Water stress inhibited ecosystem vegetation production, reduced carbon substrate supply for decomposition^51^, and induced dormancy of soil microorganisms^52^, which substantially decreased soil respiration rates. In contrast, decreased soil water content benefits diffusion of CO_2_ and O_2_, which increases the decomposition rate of soil organic matter^53^.

### Study limitations

The CRU dataset was used in this study to show the interannual variability since 1900 to quantify the frequency of the drought and heatwave event in 2013. Figure 2 shows that the CRU dataset matched very well with meteorological observations from 1950 to 2000 but underestimated the air temperature and relative humidity during the last 10 years. This gridded dataset was based on climate observations from more than 4,000 meteorological stations, with a particularly dense coverage in North America and Western Europe^54^. The study area was over the South China, and there was not sufficient sites integrated into the CRU dataset and resulted in underestimations.

This study only quantifies the immediate consequences of extreme droughts and heatwaves on carbon uptake, but the long-term impacts are likely to be significant as well because of the possible impacts on ecosystem structure (e.g., enhanced tree mortality during drought episodes), which may take a long time to recover. Although the eddy-covariance data indicate a spectacular reduction in GPP and NEP (Table 2), it is still too early to assess the impacts on the long-term carbon balance. Tree mortality is likely to occur after severe drought and can therefore result in a legacy effect on ecosystem carbon cycling 55. Moreover, changes in litterfall rates and the pool sizes of carbon reserves will have consequences beyond the duration of the extreme climate event^56^. For example, a controlled experiment showed that warming decreases NEE in both the extreme year and the following year by inducing drought that suppresses net primary productivity in the extreme year and by stimulating heterotrophic respiration of soil biota in the subsequent year^41^. Analysis of the responses to these disturbances should be conducted over the next few years.

Moreover, in this study, ecosystem models were used as a tool to investigate the effects of drought and heatwave conditions on ecosystem carbon cycling. At present, most of the models, however, do not accurately represent the responses of major ecosystem processes to climate extreme stresses yet due to our limited understanding of these processes^57^,^58^. For example, a previous study compared and evaluated four ecosystem models and found that for most sites in the Mediterranean region, models generally performed poorly, most likely because of problems in the representation of water stress effects on both carbon uptake by photosynthesis and carbon release by heterotrophic respiration^59^.

We used two ecosystem models (i.e., IBIS and EC-LUE), and both of them underestimated the impacts of drought on vegetation production (Fig. S5b). IBIS uses the Farquhar-Ball Berry type canopy photosynthesis–conductance model to simulate vegetation production, which only considers the role of stomatal conductance for limiting photosynthesis 60,61. It has become increasingly accepted that mesophyll conductance could play a role in regulating photosynthesis during periods of water stress. Keenan *et al.*^37^ adapted the Farquhar-Ball Berry type canopy photosynthesis–conductance model coupling to incorporate mesophyll conductance and found that only a combination of both mesophyll conductance and stomatal conductance limitation was successful at reproducing water stress-induced reductions in CO_2_ and water fluxes. Comprehensive photosynthesis processes need to be incorporated into the IBIS model to adequately assess the role of droughts in ecosystem carbon cycling. EC-LUE is a satellite-based model to simulate vegetation production. A recent study highlighted that all water-related variables used in LUE models have several advantages/disadvantages in terms of representing the constraint of water availability as well as the need to improve the representation of the impacts of water stress in the LUE models^28^.

Model validation showed a relatively poor performance for simulating ecosystem respiration, especially for the impacts of drought on ecosystem respiration (Fig. S4, S5c). IBIS uses a CO_2_ production model to simulate soil respiration, which is established on the principle of mass balance of carbon in ecosystems and has been developed in the past few decades to simulate terrestrial carbon processes^61^. In the CO_2_ production models, carbon allocation is still one of the difficult processes to be well represented in models^62^. When some of the fundamental relationships are largely unknown, it is beyond the reach of any process-oriented model to predict soil respiration realistically. Moreover, IBIS assumes that respiration can be described by a simple multiplicative effect of temperature and moisture, however, this assumption has been challenged. Reichstein *et al.*^63^ showed that the temperature sensitivity of respiration was not independent of moisture. It is expected that the future development of ecosystem models will include integrating comprehensive responses of ecosystem processes to climate extreme stresses, which will improve our ability to model CO_2_ and water fluxes from terrestrial ecosystems^64^.

## Additional Information

**How to cite this article**: Yuan, W. *et al.* Severe summer heatwave and drought strongly reduced carbon uptake in Southern China. *Sci. Rep.* **6**, 18813; doi: 10.1038/srep18813 (2016).

## Supplementary Material

Supplementary Information

## Figures and Tables

**Table 1:** Name, location, vegetation type, and available period of the study sites in this study.

Site code	Name	Vegetation, main genus	Latitude, longitude	Available Period	Energy closure (%)^a^	Available data (%)^b^
AJ	AnJi	Bamboo forest *Phyllostachys edulis*	30.47^°^N, 119.67^°^E	2012–2013	83	81
NX^c^	NingXiang	Evergreen broadleaf forest *Fagus longipetiolata Seem.*	28.33^°^N, 112.57^°^E	2012–2013	89	—
LA	LinAn	Bamboo forestCV.Ventricousinternode	30.30^°^N, 119.56^°^E	2012–2013	82	85
LS	LiShui	Grassland *Imperata cylindrica*	31.72^°^N, 118.98^°^E	2012–2013	91	92
HT	HuiTong	Evergreen needleleaf forest *Cunninghamia lanceolata*	26.83^°^N, 109.75^°^E	2009–2013	92	90
QYZ	QianYanZhou	Evergreen needleleaf forest *Pinus massoniana Lamb etc*	26.74^°^N, 115.06^°^E	2005–2013	90	86

^a^Averaged ratio between the sum of sensible heat (H) and latent heat (LE) and the available energy (difference of net radiation and soil heat flux).

^b^Percent of available data through the July-August.

^c^EC measurement starts on August 12 of 2012.

**Table 2:** Climate and ecosystem CO_2_ anomalies in 2013 at eddy covariance sites.

Site code	July–August						Baseline year
	ΔT	ΔP	ΔWs	ΔGPP	ΔTER	ΔNEP	
	(^o^C)	(mm)	(%)	g C m^−2^ month^−1^			
AJ	3.16	—	−38.34	−44.33	−9.92	−34.41	2012
NX	3.22	—	−11.23	−48.98	8.37	−57.35	2012
LA	3.54	—	−26.23	−46.19	−14.26	−31.93	2012
LS	1.24	−231.56	−15.23	−42.47	−19.84	−22.63	2012
HT	2.13	−125.67	−13.58	−55.49	−30.69	−24.8	2009–2012
QYZ	0.91	−43.86	−3.86	−48.98	−12.71	−36.27	2005–2012
Site average	2.37	−133.69	−18.07	−47.74	−13.02	−34.41	
Model regionalaverage	—	—	—	−52.08†	−16.74*	−35.96*	2000–2012†
				−53.01*			1960–2012*

ΔT, ΔP, ΔWs, ΔGPP, ΔTER, ΔNEP show the differences on air temperature, precipitation, soil volumetric water content, gross primary production, ecosystem respiration and net ecosystem production of July and August of 2013 with the baseline years. The smaller negative GPP, TER and NEP indicate the larger carbon release to the atmosphere. The bottom line shows the regional averaged simulations by EC-LUE and IBIS over the entire study areas (†EC`LUE simulations, *IBIS simulations).
