# Supplementary material for: Severe summer heatwave and drought strongly reduced carbon uptake in Southern China
Source: Sci Rep. 2016 Jan 7;6:18813. doi: 10.1038/srep18813 (PMC4703972; doi:10.1038/srep18813)
Supplement: Supplementary Information [file srep18813-s1.pdf]

Scientific Reports

Supporting Information for

Severe summer heatwave and drought strongly reduced carbon uptake in Southern China

Wenping Yuan[1,2*], Wenwen Cai[1], Yang Chen[1], Shuguang Liu[3], Wenjie Dong[1], Haicheng Zhang[1], Guirui Yu[4], Zhuoqi Chen[5], Honglin He[4], Weidong Guo[6], Dan Liu[1], Shaoming Liu[7], Wenhua Xiang[3], Zhenghui Xie[8], Zhonghui Zhao[3], Guomo Zhou[9]

[1]State Key Laboratory of Earth Surface Processes and Resource Ecology, Beijing Normal University, Beijing 100875, China;
[2]State Key Laboratory of Cryospheric Sciences, Cold and Arid Regions Environmental and Engineering Research Institute, Chinese Academy of Sciences, Lanzhou 730000, Gansu, China;
[3]National Engineering Laboratory for Applied Technology of Forestry & Ecology in South China, Central South University of Forestry and Technology, Changsha 410004, Hunan, China;
[4]Key Laboratory of Ecosystem Network Observation and Modeling, Synthesis Research Center of Chinese Ecosystem Research Network, Institute of Geographic Sciences and Natural Resources Research, Chinese Academy of Sciences, Beijing 100101, China;
[5]College of Global Change and Earth System Science, Beijing Normal University, Beijing 100875, China;
[6]Institute for Climate and Global Change Research & School of Atmospheric Sciences, Nanjing University, China;
[7]State Key Laboratory of Remote Sensing Science, School of Geography, Beijing Normal University, Beijing 100875, China;
[8]State Key Laboratory of Numerical Modeling for Atmospheric Sciences and Geophysical Fluid Dynamics, Institute of Atmospheric Physics, Chinese Academy of Sciences, Beijing, China;

[9]Zhejiang Agriculture and Forestry University, Lin'an 311300, China;

Contents of this file

Text S1. Introduction of EC-LUE model.

Text S2. Introduction of IBIS model

Text S3. Parameter inversion

Text S4. Climate and eddy covariance data

Figure S1 The location of eddy covariance sites, meteorology sites and study area.

Figure S2 Interannual variability of NDVI at July and August from 2000 to 2013.

Figure S3 Predicted vs. the estimated GPP (a and b), TER (c) and NEP (d) at the EC sites in Table S1. The left and right sides showed the simulations at calibration and validation sites respectively. The red lines are 1:1 line and the black lines are linear regression line.

Figure S4 Histogram of differences between simulations and observations (Sim - Obs) of gross primary production (GPP) derived from EC-LUE (a), IBIS (b), ecosystem respiration (TER) (c) and net primary production (NEP) (d). The y-axis indicates the site number. The statistics include the results of all sites (calibration and validation sites) at the Table S1.

Figure S5 Observed versus modeled difference of mean daily gross primary production (GPP) (a and b), ecosystem respiration (TER) (c) and net ecosystem production (NEP) (d). The red lines are 1:1 line and the black lines are linear regression line.

Figure S6 Regional anomalies of gross primary productivity (GPP, (a) EC-LUE model and (b) IBIS model), ecosystem respiration (TER) of IBIS (c) and net primary production (NEP) of IBIS (d) during July and August of 2013. EC-LUE model simulations compare 2013 and the average of 1982–2012, and IBIS compare 2013 and the average of 1960-2012. The unit is g C m^{-2} day^{-1}.

Table S1 Name, location, annual mean temperature, annual precipitation, and vegetation type of the study sites used for model calibration and validation.

Table S2 Calibrated model parameter values for EC-LUE and IBIS models.

[*] Wenping Yuan (yuanwpcn@126.com)

Text S1. Introduction of EC-LUE model

The EC-LUE model is a typical light use efficiency model and driven by only four variables: the normalized difference vegetation index (NDVI), photosynthetically active radiation (PAR, MJ m^{-2}), air temperature (T, ℃), and the Bowen ratio of sensible to latent heat flux (Yuan et al., 2007, 2010). The GPP was computed from the fraction of PAR absorbed by the vegetation canopy (fPAR), PAR, and down-regulators that represent plant stresses due to suboptimal temperature (T_s) and water (W_s).

$$GPP = PAR \times fPAR \times LUE_{\max} \times Min(T_s, W_s) \quad \text{(S1)}$$

$$fPAR = 1.24 \times NDVI - 0.168 \quad \text{(S2)}$$

$$T_s = \frac{(T - T_{\min}) \times (T - T_{\max})}{((T - T_{\min}) \times (T - T_{\max}) - (T - T_{opt})^2)} \quad \text{(S3)}$$

$$W_s = \frac{LE}{R_n} \quad \text{(S4)}$$

where LUE_{max} is the potential light use efficiency without environmental stress (g C m^{-2} MJ^{-1} APAR). Min denotes the minimum values of T_s and W_s, and we assumed that the impacts of temperature and moisture on LUE follow Liebig's Law (i.e., LUE is only affected by the most limiting factor at any given time). T_{min}, T_{max} and T_{opt} are the minimum, maximum and optimum air temperatures (℃) for photosynthetic activity, respectively. If air temperature falls below T_{min} or increases beyond T_{max}, T_s is set to zero. In this study, T_{min} and T_{max} were set to 0 and 40℃, respectively (Yuan et al., 2007). LE is latent heat (MJ m^{-2}), which is estimated by the revised RS-PM (Remote Sensing-Penman Monteith) model (Yuan et al., 2010a). R_n is net radiation (MJ m^{-2}). LUE_{max} and T_{opt} were two parameters, and determined using nonlinear optimization.

Text S2. Introduction of IBIS model

Integrated BIosphere Simulator (IBIS) is hierarchically organized to allow explicit coupling among ecological biophysical and physiological processes at different timescales (Foley et al., 1996). IBIS uses a mechanistic Farquhar model for the treatment of canopy photosynthesis (Farquhar et al., 1980). The CO_2 production models are established for ecosystem respiration processes on the principle of mass balance of carbon in ecosystems, which generally share a common structure that partitions carbon input into several pools from which carbon is released via respiratory processes. For spatially explicit simulations of carbon and water fluxes, MODIS land cover product (MOD12) was combined with Köppen-Geiger climate classification map to derive the plant function type distribution over the study areas, and which were held constant.

The gross photosynthesis rate per unit leaf area, A_g (mol CO_2 m^{-2} s^{-1}) was expressed as

$$A_g = \min(J_e, J_c, J_s) \qquad \text{(S5)}$$

where J_e is light-limited rate of photosynthesis, J_c represents the Rubisco-limited rate of photosynthesis, and J_s is the photosynthesis limited by the inadequate rate of utilization of triose phosphate.

The light-limited rate of photosynthesis is given as

$$J_e = \alpha_3 Q_p \frac{C_i - \Gamma_*}{C_i + 2\Gamma_*} \qquad \text{(S6)}$$

where α_3 is the intrinsic quantum efficiency for CO_2 uptake in C_3 plants (mol CO_2 mol^{-1} quanta), Q_p is the flux density of photosynthetically active radiation absorbed by the leaf

(mol quanta m^{-2} s^{-1}), C_i is the concentration of CO_2 in the intercellular air spaces of the leaf (mol mol^{-1}), and Γ_* is the compensation point for gross photosynthesis (mol mol^{-1}).

The Rubisco-limited rate of photosynthesis is calculated as

$$J_c = \frac{V_m(C_i - \Gamma_*)}{C_i + K_c(1 + \frac{[O_2]}{K_o})} \quad \text{(S7)}$$

where V_m is the maximum carboxylase capacity of Rubisco (mol CO_2 m^{-2} s^{-1}), and K_c and K_o are the Michaelis-Menten coefficients (mol mol^{-1}) for CO_2 and O_2, respectively. The parameter V_m (mol CO_2 m^{-2} s^{-1}) is an important parameter for simulating plant photosynthesis process. In the IBIS model, V_{m15} is set as an undetermined model parameter, which indicates the maximum carboxylase capacity of Rubisco at 15℃ and without water stress. Then, the following equation is used to simulate actual V_m by considering the reduction of carboxylase capacity under limiting environmental conditions:

$$V_m = V_{m15} \times T_s \times W_s \quad \text{(S8)}$$

$$T_s = \frac{\exp(3500 \times (0.00347 - (1/(T_l + 273.16))}{(1 + \exp(0.5 \times (5 - T_l)) \times (1 + \exp(0.5 \times (T_l - 25))} \quad \text{(S9)}$$

$$W_s = \sum_{i=1}^{n} \frac{SWC_i - Swilt_i}{Sfield_i - Swilt_i} \times Root_i \quad \text{(S10)}$$

where T_s and W_s are the temperature and water scalar for carboxylase capacity of Rubisco. T_l is leaf surface temperature (℃), and T_s shows a increasing exponentially trend with temperature in its low range, reaching a maximum at an optimal temperature (20℃), and then declining. SWC_i, $Swilt_i$ and $Sfield_i$ indicate the volumetric soil moisture content, soil wilt point and field point at i*th* layer of soil. $Root_i$ indicate the total root biomass in i*th* soil layer following Jackson et al. (1996).

Under conditions of light intercellular CO_2 concentrations and high irradiance, photosynthesis was limited by the inadequate rate of utilization of triose phosphate. This limitation was expressed as

$$J_s = 3T\left(1 - \frac{\Gamma_*}{C_i}\right) + \frac{J_p \Gamma_*}{C_i} \tag{S11}$$

where T is the rate of triose phosphate utilization.

Soil organic matter decomposition is simulated using a daily time step. Following Parton et al. (1987) and Verberne et al. (1990), the main partitioning of carbon in IBIS is between surface and belowground carbon litter pools derived from litterfall (leaf turnover, woody detritus, and fine root turnover) and the more recalcitrant soil organic matter pools. Annual litterfall and fine root turnover from the previous year are incorporated into the litter pools.

The detritus is divided between decomposable (DPM, decomposable plant matter), structural (SPM, structural plant matter), and lignified (RPM, resistant plant matter) fraction. The base decay rates of litter, root, and soil carbon and microbial biomass turnover are modified by functions of soil temperature and soil moisture. In the IBIS model, the base decay rates of all components are given. In this study, we selected the decay rate of leaf DPM as undetermined parameter (Dr). Modifying factors are used to simulate the temperature and moisture effects on microbial activity. The Arrhenius equation, based on the kinetic theory, is used to characterize impacts of decay rates with temperature (Lloyd and Taylor 1994):

$$T_f = e^{E_0 \times \left(\frac{1}{T_{ref} - T_0} - \frac{1}{T_s - T_0}\right)} \tag{S12}$$

where T_s is the absolute soil temperature in degrees Kelvin (K), T_{ref} is a reference temperature in degrees Kelvin (K) (288.16 K), E_0 is an activation-energy-type empirical

coefficient, and T_0 is the low temperature limit for the soil respiration in Kelvin (K) (227.14 K; Lloyd and Taylor, 1994). E_0 is an important model parameter to determine.

Equation (S13) is used to describe decay rate with soil moisture (Friend et al., 1995):

$$W_f = \begin{cases} (0.000371 \times wfps^2 - 0.0748 \times wfps) + 4.13, wfps \geq 60 \\ e^{(wfps-60)^2/-800}, wfps < 60 \end{cases} \quad \text{(S13)}$$

where wfps indicate relative soil moisture content (the ratio of volumetric soil water content and total porosity).

Text S3. Parameter inversion

In this study, MCMC (Markov chain Monte Carlo) procedure was used to inverse model parameter in this study, and the Metropolis-Hastings (M-H) algorithm was used as the MCMC sampler (Xu et al., 2006; Yuan et al., 2012). The optimal V_{m15}, E_0 and D_r of IBIS and LUE_{max} and T_{opt} of EC-LUE were obtained to be parameter values for each vegetation type.

Text S4. Climate and eddy covariance data

We simulated the changes in carbon fluxes from 1960-2013 using an interpolated climate data. The thin plate smoothing splines method was used to generate the daily interpolated mean air temperature, maximum and minimum temperature, relative humidity, precipitation, wind speed, sun shine hours and atmospheric pressure for the study area at a spatial resolution of 25 kilometers of latitude and longitude for the period of 1960-2013. The fitted trivariate splines incorporated a spatially varying dependence on ground elevation and automatically adapted to the large variation in

station density throughout China. A comprehensive introduction to the technique of thin plate smoothing splines is given in Wahba (1990). Moreover, an Angstrom type correlation method was used to calculate downward solar radiation (R_g) from the sunshine duration data (Almorox et al., 2004), and R_g was transferred to photosynthetically active radiation (PAR) with the ratio of 0.5. We conducted 1,000-year spin-up before 1960 repeatedly using Climatic Research Unit Global Climate Dataset (CRU3.10) over the period 1900-1959, which provides monthly mean climate variables at 0.5×0.5°.

We used the LaThuile FLUXNET dataset (http://www.fluxdata.org) to evaluate model performance. In total, sixty-three eddy covariance (EC) flux tower sites were included in this study (Table S1), from six major terrestrial biomes: deciduous broadleaf forest, evergreen broadleaf forest, evergreen needleleaf forest, mixed forest, grassland and shrubland. These selected sites show the similar climate condition (i.e. mean annual temperature and annual precipitation) with the study area. Twenty-eight sites were randomly selected to calibrate the EC-LUE and IBIS models, and other thirty-three sites were used to validate models. Six EC sites within the study area were only used to examine models (Table S1). The calibrated parameters for EC-LUE and IBIS were showed at Table S2. Fig. S3 shows the model performance to simulate the magnitude of GPP, TER and NEP at the calibration and validation sites.

In order to examine the model performance for simulating the impacts of drought, we selected 10 eddy covariance sites where experienced the severity drought of 2003 at the European (Table S1) (Ciais et al., 2005). Moreover, six eddy covariance sites over the study area were used to examine the model ability for simulating the impacts of

drought on carbon fluxes. We compared the observed and simulated differences of GPP, TER and NEP between 2002 and 2003. The results showed that IBIS can simulate the impacts of drought very well (Fig. S6).

References

Almorox, J., and Hontoria, C. Global solar radiation estimation using sunshine duration in Spain. Energy Conversion and Management 45, 1529-1535 (2004).

Ciais, P. *et al*. Europe-wide reduction in primary productivity caused by the heat and drought in 2003. Nature 437, 529-533 (2005).

Farquhar, G.D., von Caemmerera, S., and Berry, J.A. A biogeochemical model of photosynthetic CO_2 assimilation in leaves of C3 species. Planta 149, 78-90 (1980).

Foley, J.A. *et al*. An integrated biosphere model of land surface processes, terrestrial balance and vegetation dynamics. Global Biogeochemical Cycles 10, 603-628 (1996).

Friend, A.D., Stevens, A.K., Knox, R.G., and Cannell, M.G.R. A process-based terrestrial biosphere model of ecosystem dynamics (Hybrid v3.0). Ecological Modelling 95, 249-287 (1995).

JacksonR, B., Canadell, J., and Ehleringer J.R. A global analysis of root distribution of terrestrial biomes. Oecologia 108, 389-411 (1996).

Lloyd, J., and Taylor J.A. On the temperature dependence of soil respiration, Functional Ecol. 8, 315-323 (1994).

Parton, W.J., Schimel, D.S., Cole, C.V., and Ojima, D.S. Analysis of factors controlling soil organic matter levels in Great Plains grasslands. Soil Science Society of America Journal 51, 1173-1179 (1987).

Verberne, E.L. *et al*. Modeling organic matter dynamics in different soils. Netherlands Journal of Agricultural Science 38, 221-238 (1990).

Wahba, G. Spline models for observational data. CBMSNSF Regional Conference Series in Applied Mathematics. 59, Society for Industrial and Applied Mathematics, 169 (1990).

Xu, T., White, L., Hui, D.F., and Luo, Y. Q. Probabilistic inversion of a terrestrial ecosystem model: analysis of uncertainty in parameter estimation and model prediction. Global Biogeochemical Cycles 20, GB2007 (2006).

Yuan, W.P. *et al*. Global estimates of evapotranspiration and gross primary production based on MODIS and global meteorology data. Remote Sensing of Environment 114, 1416-1431 (2010).

Yuan, W.P. *et al*. Deriving a light use efficiency model from eddy covariance flux data for predicting daily gross primary production across biomes. Agricultural and Forest Meteorology 143, 189-207 (2007).

Yuan, W.P. *et al*. Improving model parameter estimation using coupling relationships between vegetation production and ecosystem respiration. Ecological Modelling 240, 29-40 (2012).

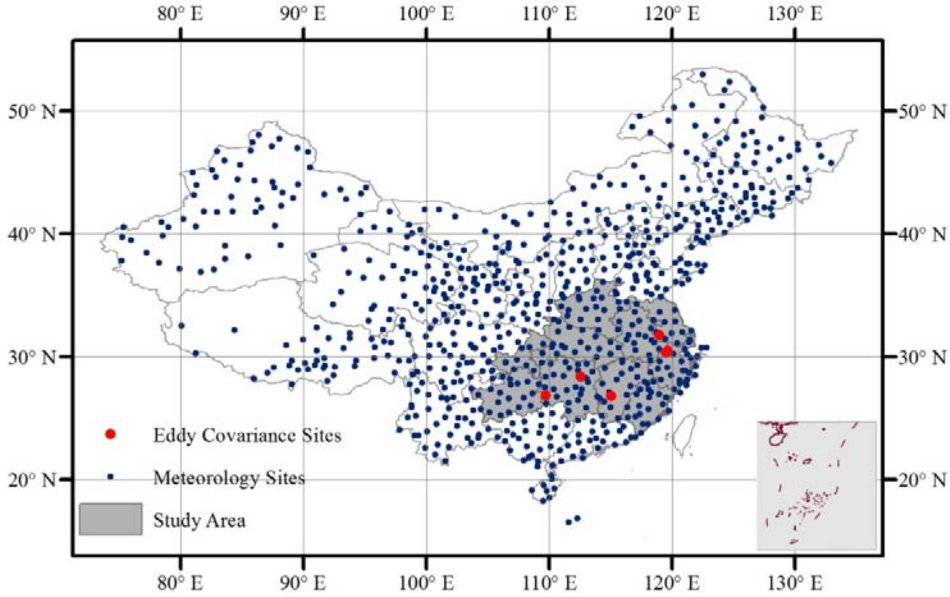

Figure S1 The location of eddy covariance sites, meteorology sites and study area. The maps were created by the ArcMap 9.3.

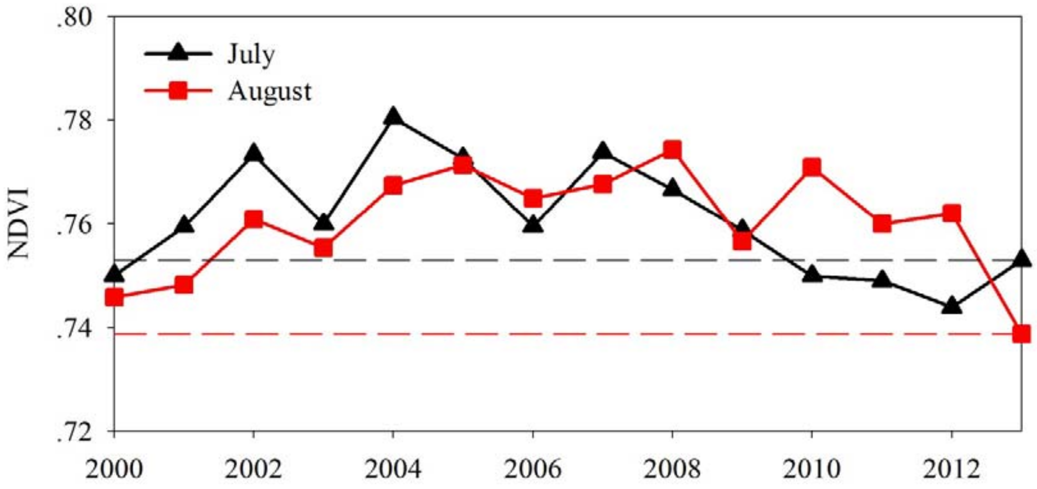

Figure S2 Interannual variability of NDVI at July and August from 2000 to 2013.

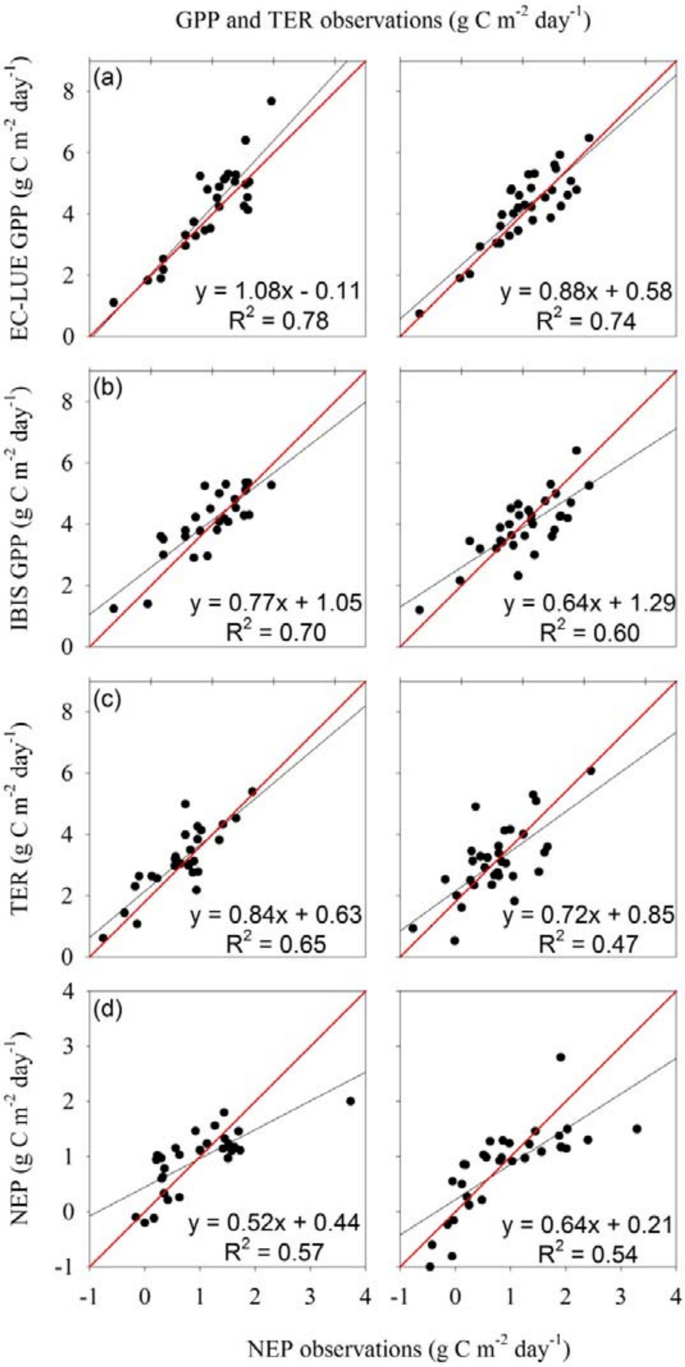

Figure S3 Predicted vs. the estimated GPP (a and b), TER (c) and NEP (d) at the EC sites in Table S1. The left and right sides showed the simulations at calibration and validation sites respectively. The red lines are 1:1 line and the black lines are linear regression line.

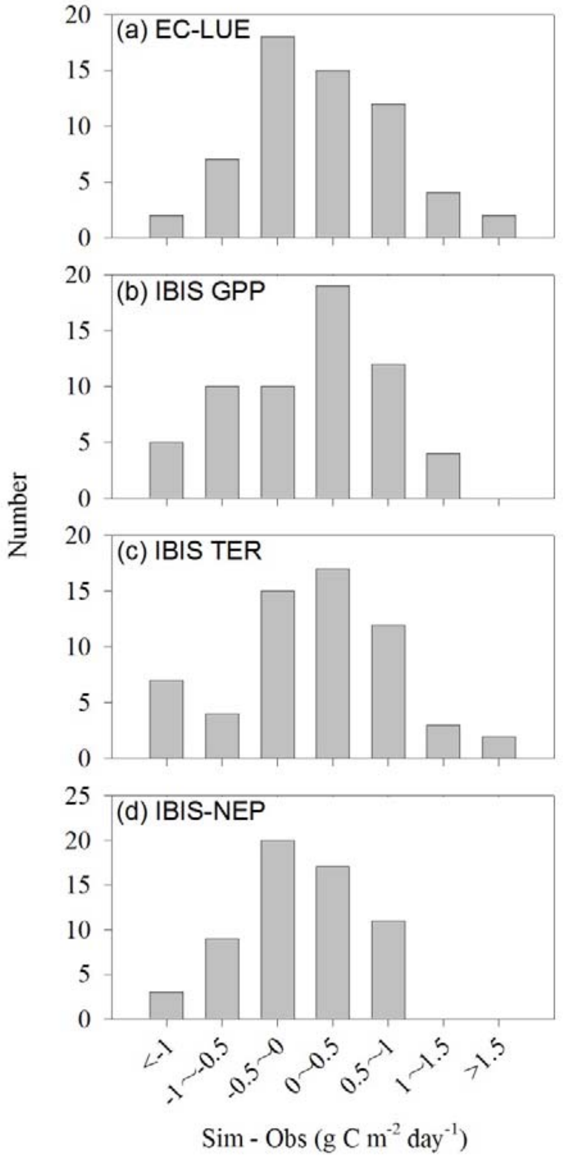

Figure S4 Histogram of differences between simulations and observations (Sim - Obs) of gross primary production (GPP) derived from EC-LUE (a), IBIS (b), ecosystem respiration (TER) (c) and net primary production (NEP) (d). The y-axis indicates the site number. The statistics include the results of all sites (calibration and validation sites) at the Table S1.

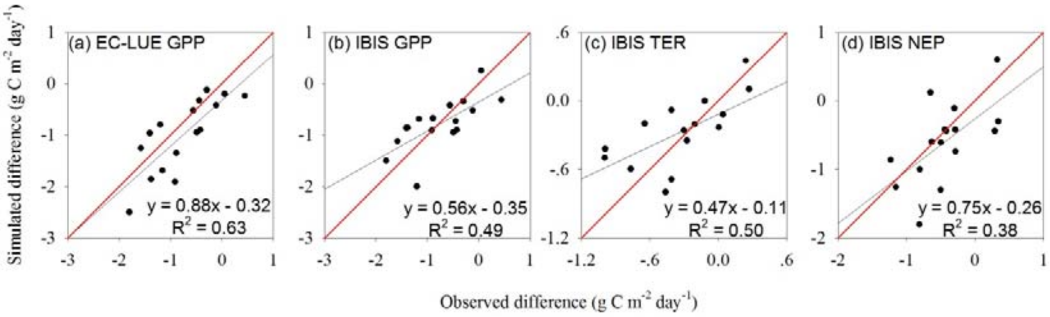

Figure S5 Observed versus modeled difference of mean daily gross primary production (GPP) (a and b), ecosystem respiration (TER) (c) and net ecosystem production (NEP) (d). The red lines are 1:1 line and the black lines are linear regression line.

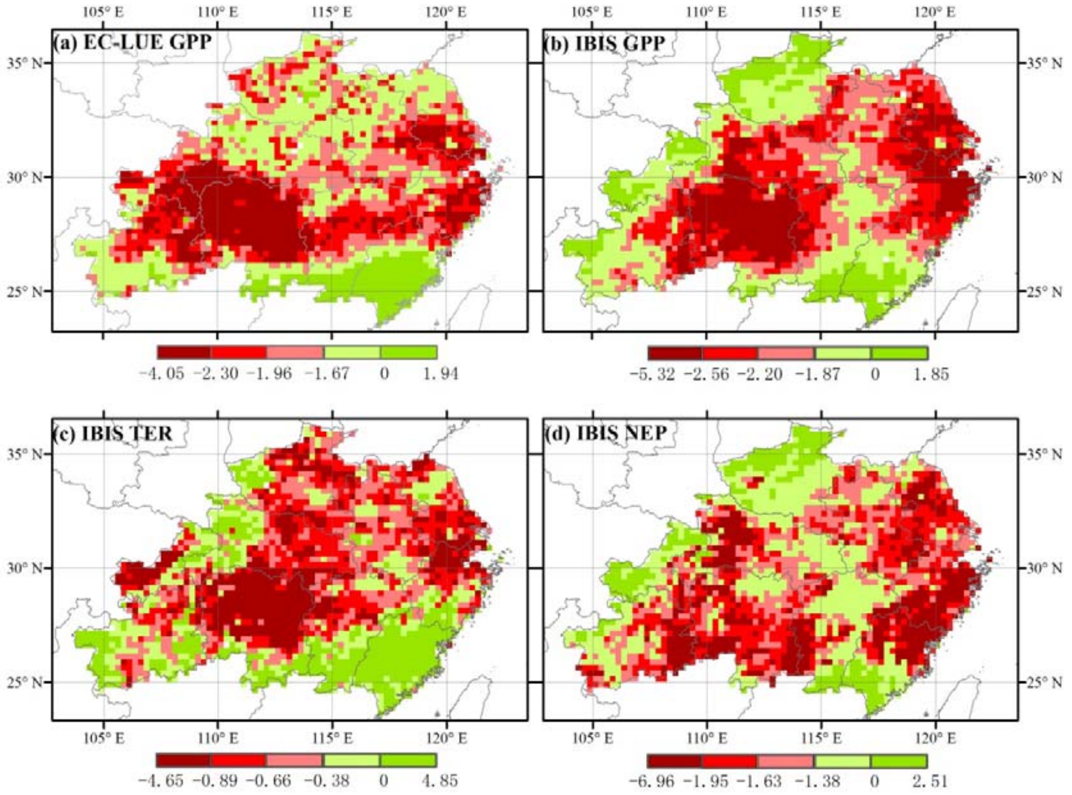

Figure S6 Regional anomalies of gross primary productivity (GPP, (a) EC-LUE model and (b) IBIS model), ecosystem respiration (TER) of IBIS (c) and net primary production (NEP) of IBIS (d) during July and August of 2013. EC-LUE model simulations compare 2013 and the average of 1982–2012, and IBIS compare 2013 and the average of 1960-2012. The unit is g C m^{-2} day^{-1}. The maps were created by the ArcMap 9.3.

Table S1 Name, location, annual mean temperature, annual precipitation, and vegetation type of the study sites used for model calibration and validation.

**Site[1]**	**Latitude**	**Longitude**	**Vegetation type[2]**	**AMT (℃)[3]**	**AMP (mm yr^{-1})[4]**
Model calibration sites					
DK-Sor*	55.49°N	11.65°E	DBF	6.54	611.23
FR-Hes*	48.67°N	7.06°E	DBF	4.95	942.69
IT-Non	44.69°N	11.09°E	DBF	5.81	809.18
IT-Ro1*	42.41°N	11.93°E	DBF	12.00	700.00
UK-Ham	51.15°N	0.86°W	DBF	7.59	848.36
US-Ha1	42.54°N	72.17°W	DBF	2.71	1171.34
US-MOz	38.74°N	92.20°W	DBF	7.39	1004.63
FR-Pue*	43.74°N	3.60°E	EBF	9.60	667.33
IT-Lec	43.30°N	11.27°E	EBF	11.44	819.28
PT-Mi1	38.54°N	8.00°W	EBF	12.79	729.76
DE-Tha*	50.96°N	13.57°E	ENF	3.66	575.27
ES-ES1*	39.35°N	0.32°W	ENF	9.58	390.83
IT-Lav	45.96°N	11.28°E	ENF	7.97	766.45
NL-Loo	52.17°N	5.74°E	ENF	5.71	785.63
US-Blo	38.90°N	120.63°W	ENF	9.04	471.46
US-SP3	29.75°N	82.16°W	ENF	15.35	1373.51
DE-Meh	51.28°N	10.66°E	GRA	4.07	666.32
ES-VDA	42.15°N	1.45°E	GRA	9.32	537.87
FR-Lq2	45.64°N	2.74°E	GRA	4.88	915.61
HU-Mat	47.85°N	19.73°E	GRA	5.27	525.34
IT-MBo	46.02°N	11.05°E	GRA	3.39	1110.22
NL-Ca1	51.97°N	4.93°E	GRA	6.55	805.12
NL-Hor	52.03°N	5.07°E	GRA	5.94	830.91
UK-EBu	55.87°N	3.21°W	GRA	4.25	1483.73
US-Aud	31.59°N	110.51°W	GRA	6.82	445.34
BE-Bra	51.31°N	4.52°E	MF	6.30	858.54
BE-Vie*	50.31°N	6.00°E	MF	4.41	1187.08
US-SP2	29.76°N	82.24°W	OSH	15.35	1373.51
Model validation sites					
DE-Hai*	51.08°N	10.45°E	DBF	4.12	725.47
FR-Fon	48.48°N	2.78°E	DBF	7.01	649.37
IT-Col	41.85°N	13.59°E	DBF	11.24	785.67
IT-PT1	45.20°N	9.06°E	DBF	8.39	1069.57
IT-Ro2	42.39°N	11.92°E	DBF	12.00	700.00
UK-PL3	51.45°N	1.27°W	DBF	6.54	853.87
US-MMS	39.32°N	86.41°W	DBF	6.35	1165.92
US-Oho	41.55°N	83.84°W	DBF	4.17	903.63
IT-Cpz	41.71°N	12.38°E	EBF	11.00	700.00
PT-Esp	38.64°N	8.60°W	EBF	12.56	1000.21
CZ-BK1	49.50°N	18.54°E	ENF	2.34	965.85
DE-Wet	50.45°N	11.46°E	ENF	4.29	608.57
FR-LBr*	44.72°N	0.77°W	ENF	9.74	1180.52

IT-SRo	43.73°N	10.28°E	ENF	11.07	1041.87
UK-Gri	56.61°N	3.80°W	ENF	3.58	2036.28
US-SP1	29.74°N	82.22°W	ENF	15.35	1373.51
DE-Gri	50.95°N	13.51°E	GRA	3.66	575.27
DK-Lva	55.68°N	12.08°E	GRA	6.05	582.99
FR-Lq1	45.64°N	2.74°E	GRA	4.88	915.61
HU-Bug	46.69°N	19.60°E	GRA	5.85	552.97
IT-Amp	41.90°N	13.61°E	GRA	11.24	785.67
IT-Mal	46.12°N	11.70°E	GRA	3.72	1035.65
NL-Haa	52.00°N	4.81°E	GRA	6.57	821.90
PT-Mi2	38.48°N	8.02°W	GRA	12.71	688.95
UK-Tad	51.21°N	2.83°W	GRA	6.81	930.94
US-Goo	34.25°N	89.87°W	GRA	10.84	1381.11
BE-Jal	50.56°N	6.07°E	MF	4.70	1058.07
IT-Pia*	42.58°N	10.08°E	OSH	12.14	702.64
Model validation sites at the study area					
AJ	30.47°N	119.67°E	DBF	16.10	1344
NX	28.33°N	112.57°E	EBF	16.80	1358
LA	30.30°N	119.56°E	DBF	15.80	1453
LS	31.72°N	118.98°E	GRA	15.40	1062
HT	26.83°N	109.75°E	ENF	16.70	1780
QYZ	26.74°N	115.06°E	ENF	17.90	1542

[1]Sites with star mark also were used to examine the model performance for simulating the impacts of drought.
[2]Vegetation type: DBF (deciduous broadleaf forest), EBF (evergreen broadleaf forest), ENF (evergreen needleleaf forest), GRA (grassland), MF (mixed forest) and OSH (shrubland). [3]AMT: annual mean temperature. [4]AMP: annual mean precipitation.

Table S2 Calibrated model parameter values for EC-LUE and IBIS models.

Vegetation type	IBIS			EC-LUE	
	V_{m15}	E_0	D_r	LUE_{max}	T_{opt}
DBF	42	206	0.11	2.28	16.25
EBF	35	211	0.10	2.13	19.87
ENF	29	236	0.16	2.03	15.02
GRA	26	206	0.09	1.95	17.58
MF	36	195	0.08	2.16	17.23
OSH	39	203	0.16	2.26	16.58
CRP	40	206	0.12	2.03	15.63

Vegetation type: DBF (deciduous broadleaf forest), EBF (evergreen broadleaf forest), ENF (evergreen needleleaf forest), GRA (grassland), MF (mixed forest), OSH (shrubland) and CRP (cropland). V_{m15}: maximum Rubiscoc arboxylation capacity at 15℃ (10^{-6} mol CO_2 m^{-2} s^{-1}). E_0: activation-energy-type empirical coefficient. Dr: adjusted coefficient for litterfall decomposition. LUE_{max}: potential light use efficiency (g C MJ^{-1}). T_{opt} is optimal temperature for plant photosynthesis (℃).
